# The Genomic and Morphological Effects of Bisphenol A on *Arabidopsis thaliana*

**DOI:** 10.1371/journal.pone.0163028

**Published:** 2016-09-15

**Authors:** Derek Frejd, Kiera Dunaway, Jennifer Hill, Jesse Van Maanen, Clayton Carlson

**Affiliations:** Department of Biology, Trinity Christian College, Palos Heights, Illinois, United States of America; National Taiwan University, TAIWAN

## Abstract

The environmental toxin bisphenol A (BPA) is a known mammalian hormone disrupter but its effects on plants have not been well established. The effect of BPA on gene expression in *Arabidopsis thaliana* was determined using microarray analysis and quantitative gene PCR. Many hormone responsive genes showed changes in expression after BPA treatment. BPA disrupted flowering by a mechanism that may involve disruption of auxin signaling. The results presented here indicate that BPA is a plant hormone disrupter.

## Introduction

Bisphenol A (4,4'-(propane-2,2-diyl)diphenol), or BPA, is a high production volume chemical used in the manufacture of polycarbonate plastics, epoxy resins, and other products. According to the Environmental Protection Agency, more than 1,100,000lbs of BPA are released into the environment each year[1]. BPA is an environmental contaminant that was among the 5 most commonly found organic waste water contaminants in 47 ground water sites across the United States with a maximum concentration of 2.55μg/L, (11nM)[2]. BPA is detectable at levels above 1μg/L (4.3nM) in treated waste water from water treatment facilities and is present at much higher concentrations in wastewater sludge, including sludge used in agriculture[3–5]. The effect of BPA on mammals continues to be studied closely. BPA is a reproductive and developmental toxicant in research animals (rats, primates), and may disrupt estrogen signaling causing endocrine-related neurotoxicological effects and delayed puberty [6,7]. Response to BPA in aquatic and soil dwelling animals is being investigated[8,9]. However, the effect of BPA on plants has not been thoroughly studied. Estrogens have been shown to disrupt the activity of the plant hormone auxin[10] and BPA could have a similar effect.

A study by Staples *et al*. calculated the predicted exposure concentrations of BPA in soil based on waste water sludge levels and standard agricultural use of the sludge as fertilizer[8]. Six different plant species (cabbage, corn, oats, soybeans, tomatoes, and wheat) were tested for defects in germination after exposure to high levels of BPA (up to 800mg/kg of dry weight). They found reduced germination only in 2 species and only at concentrations above 320mg/kg. Another study highlighted the ability of BPA to disrupt mitosis and microtubule formation in young pea plants[11]. The plant roots were exposed to concentrations of BPA from 20 to 50mg/L. In a separate study that also analyzed the effect of BPA at high concentrations, Speranza and others tested the effect of 10–50 mg/L BPA to induce expression of stress response proteins[12]. They found that, in kiwi fruit pollen, the stress response protein BiP (Binding Protein) was expressed in a BPA dose dependent manner. In mung bean seedlings BPA may disrupt nucleic acid levels[13]. There is some evidence that BPA may act as a cytokinin in *A*. *thaliana*[14]. Roots of *A*. *thaliana* exposed to 0.1μg/L for as little as 30min show increased expression of two *Arabidopsis* response regulators (ARRs), ARR15 and ARR16[15]. These genes did not respond to BPA in a concentration-dependent manner. At high concentrations (13μg/L) BPA resulted in decreased expression of genes related to photosynthesis in *Arabidopsis* seedlings[16]. The broader effect of low level BPA on gene expression across the genome has not yet been determined.

The goal of this project is to measure the effect of BPA on gene expression in *A*. *thaliana* and to determine the consequences of those changes. To measure the effect of BPA on whole genome expression in *A*. *thaliana*, plants were grown for 14 days after germination and then treated with 25mL of 0.1μg/L BPA solution in water for 14 days. Total RNA was collected and tested on an *A*. *thaliana* 385k Roche Nimblegen Gene Expression Microarray. Numerous genes had changes in gene expression in response to BPA, including many involved in hormone response. The changes were concentration-dependent and disrupted the plants’ ability to respond to the hormone auxin.

## Methods

### Strain and growth conditions

For the genomic analysis, square 7.6cm pots were filled ⅔ full of soilless starter mix which consisted of sphagnum peat moss, vermiculite, small limestone pieces, and a wetting agent. Another sprinkling of vermiculite was added to the top layer of soil. The pots were divided evenly into treatment and control groups, but placed randomly into trays to ensure equal treatment during the initial germination and developmental process. Each pot received 4–6 Columbia, Col-0, seeds (Lehle) of *A*. *thaliana*. The pots were misted from above until the soilless mixture was saturated and then sub-irrigated with approximately 1 cm of water. The plants were allowed to grow under full spectrum lights 14h per day with sub-irrigation for 12 days post germination. Beginning on the 13th day control plants received 25mL of water each day, applied via spray from overhead at close range. Treated plants were sprayed with water containing 0.1μg/mL of BPA unless otherwise noted.

In order to confirm that the changes in gene expression observed are a response to BPA, a concentration gradient experiment was performed. Plants were grown in starter mix as above and treated daily with a BPA solution (0nM, 10nM, 100nM, 1μM, or 10μM).

For the auxin response, sterile seeds were grown on Murashige and Skoog Basal Salt Mixture and MES media in sterile jars under 24h full spectrum light. Four sets of plants were exposed to 0, 10, 100, 1,000, or 10,000nM BPA and 0, 3, 10, 33μM indole-3-acetic acid (auxin). After one month, the number of stalks and flowers per jar were measured and the total plant material per jar was weighted. The average and standard error of the four jars for each condition were calculated.

### RNA analysis

RNA was harvested from the plants using the Qiagen RNeasy Plant Mini Kit following the manufacturer’s directions. Microarray analyses were performed by Ambry Genetics. RNA quality and concentration were determined on the Agilent Bioanalyzer and NanoDrop spectrophotometer. Double-stranded cDNA was synthesized using Roche cDNA Synthesis System. One color labeling reactions were prepared using the Roche NimbleGen Gene Expression protocol (Version 5.1) with 5μg total RNA input. The 5μg of total RNA was converted into double-stranded cDNA using an oligo (dT) primer. The double-stranded cDNA was then labeled with Cy-3 Random-Nonamers by Exo-Klenow fragment. Labeled cDNA was purified by ethanol precipitation, and the labeling efficiency was determined by the Nanodrop spectrophotometer. Next, 6μg of labeled cDNA were prepared for hybridization and placed on the *A*. *thaliana* 385k Roche Nimblegen Gene Expression Microarray and hybridized for 16–20 hours at 42°C using a Maui hybridization system. Finally, the arrays were washed and scanned at 5μM resolution on a Nimblegen MS 200 High Resolution Scanner.

Microarray results for some genes were confirmed by quantitative PCR. cDNA was produced using iScript cDNA Synthesis Kit (BioRad). The 40μL reaction included 8μL of iScript reaction mix, 2μL of iScript Reverse Transcriptase, and 0.5 to 1μg of total RNA. The reaction was incubated at 42°C for 90min and 85°C for 5min. cDNA was quantified by nanodrop on a BioTek Take3 plate.

**Fig 1:**
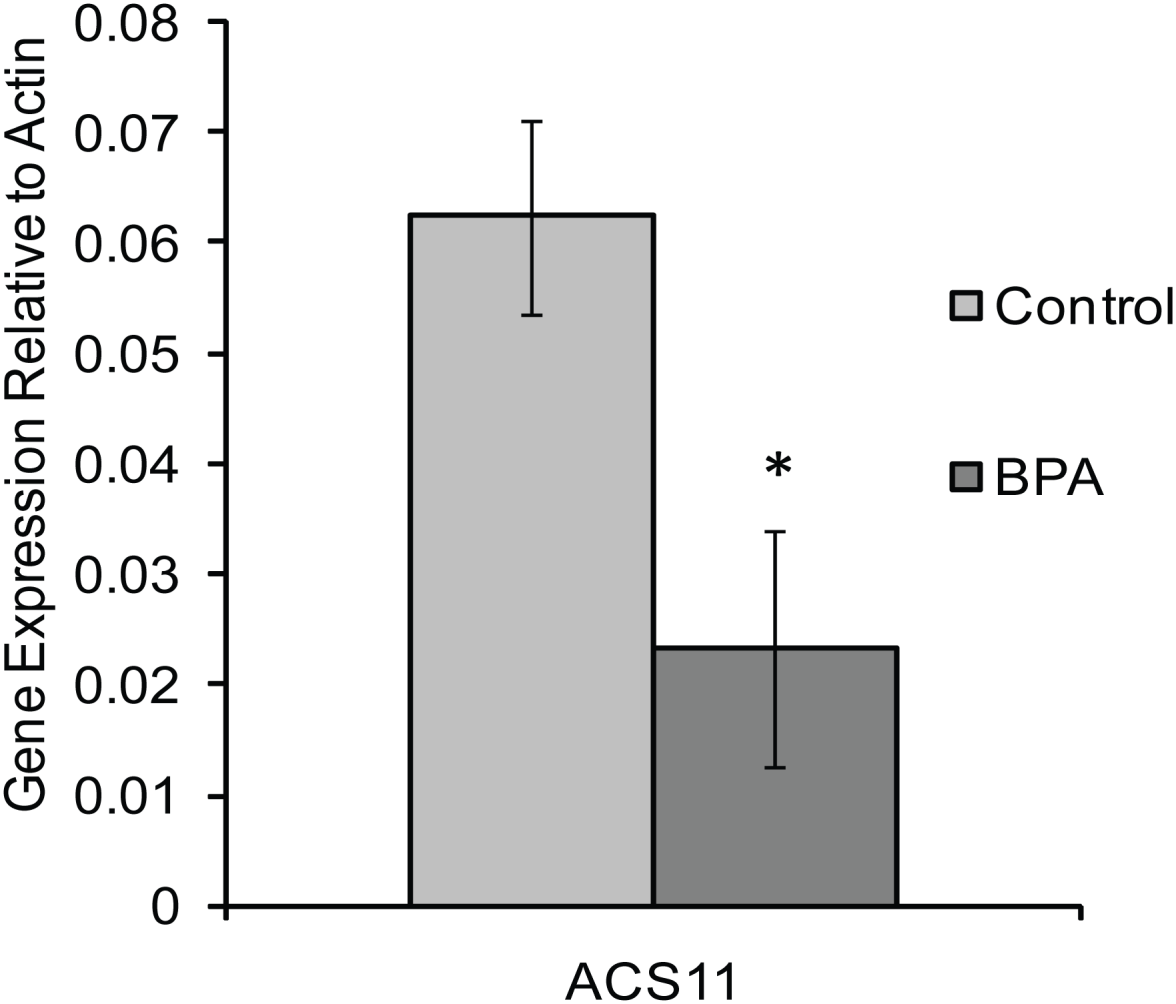
BPA changes gene expression of *ACS11*. The hormone responsive gene *ACS11* shows decreased gene expression upon treatment with 200nM BPA once daily for 28 days. Bar represents average of three technical replicates from pooled data of 20 pots of plants. Error bars represent standard error. * indicates p-values <0.05 when compared to 0nM BPA plants.

**Fig 2:**
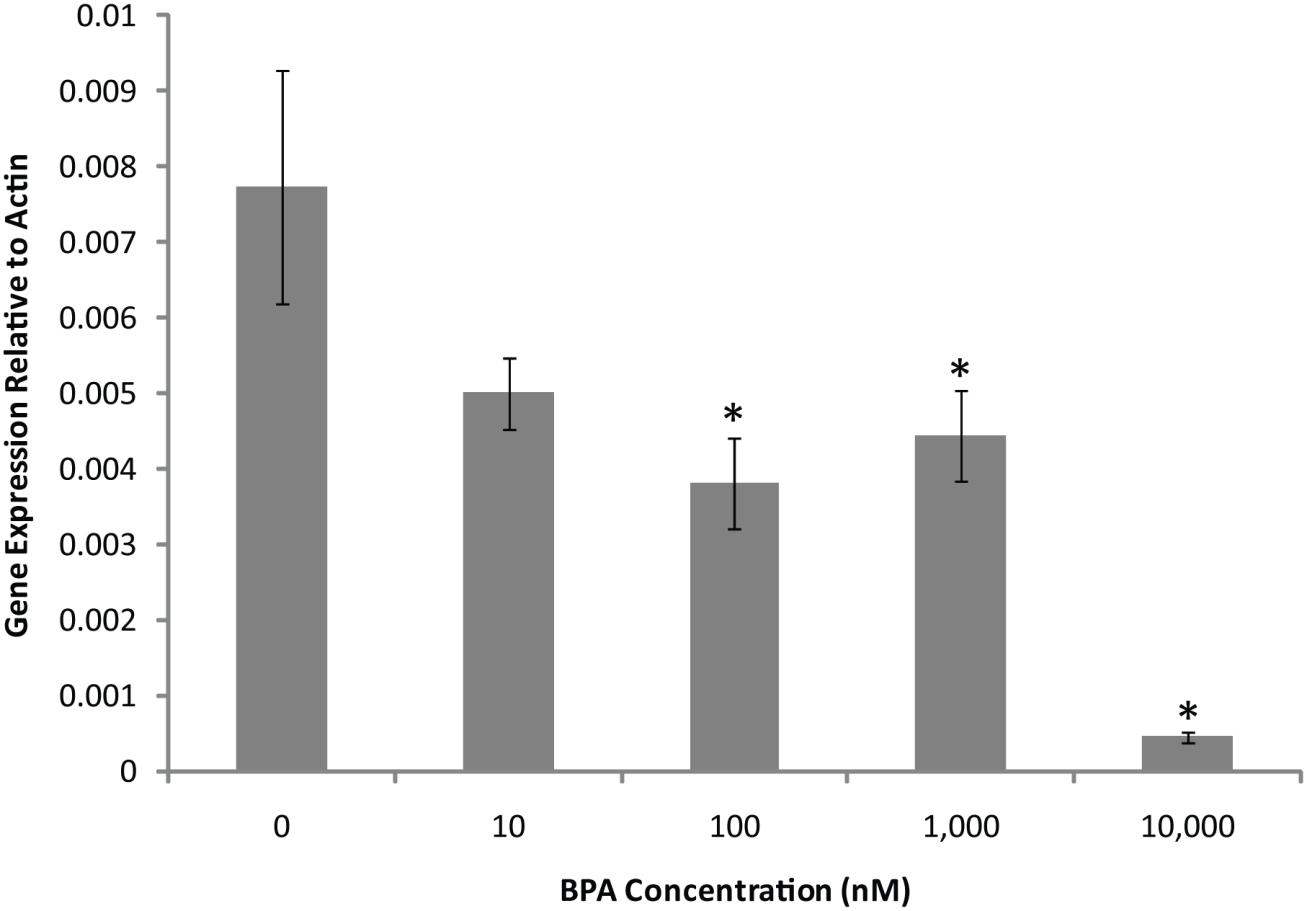
Changes in *ACS11* gene expression were dependent on BPA concentration. Higher concentrations of BPA showed stronger depression of *ACS11* expression. Each bar represents pooled data from 20 pots treated with BPA daily for 28 days. Average is from 8 technical replicates (7 for the 10nM data). Error bars show standard error. * indicates p-values <0.05 when compared to 0nM BPA plants.

Real-time PCR was performed using the iQ SYBR kit (BioRad) on an MJ Mini Personal thermal cycler equipped with the Mini Opticon RT PCR system, and each sample was analyzed 3 to 5 times. The reaction volume included 2 μL of RT product, 25μL of 2× iQ SYBR Master Mix, and 1.5μL of primer (forward and reverse, 10μM each). Actin2 (At3g18780) was amplified using primers from Sigma (C3615) and *ACS11* (At4g08040) was amplified using primers designed with AtRTPrimer[17] and synthesized by Integrated DNA Technologies (Forward = 5’-GGAGATGCCTTTCTTATCCCTGCAC and Reverse = 5’-TGTACCCATTTGAGCTTACGCAATG). The reactions were incubated at 95°C for 3 min, followed by 40 cycles of 95°C for 15s, 65°C for 45s, and 72°C for 60s. The relative change in RNA expression upon BPA treatment was measured using ΔΔCt. The ΔCt was calculated by subtracting the Ct value of Actin from the Ct value of *ACS11*. The relative gene expression was calculated using the equation 2^(−ΔΔCt). Average gene expression and standard error of gene expression were calculated for 3 replicates in Fig 1 and 8 replicates in Fig 2.

### Microarray Analysis

Data were processed through NimbleScan 2.6 using the 2006-08-28_Arabidopsis_60mer_expr design files. All samples passed QC metrics set forth by Roche NimbleGen. Normalized expression values for BPA treatment per control were calculated. Functional annotation of genes up, down, or showing disrupted regulation (either up or down) was determined by DAVID (Database for Annotation, Visualization and Integrated Discovery) analysis with the A. thaliana genome background[18]. Microarray data is available from ArrayExpress (https://www.ebi.ac.uk/arrayexpress/).

## Results, Discussion, Conclusions

### BPA disrupts expression of genes involved in hormone signaling

To determine the effects of BPA on gene expression in *A*. *thaliana*, microarray analysis was performed on BPA treated and control plants. Analysis of the transcriptome of both samples identified 108 genes with disrupted gene expression in response to the toxin. Four genes showed greater than 2-fold increase and 41 genes had greater than 1.5-fold increase in expression, while 67 genes presented with less than 0.5-fold expression after treatment. DAVID analysis revealed significant disruption of genes involved in response to hormone stimulus (Table 1). The 22 genes involved in hormone stimulus that showed a change in gene expression in response to BPA included 4 small auxin upregulated RNAs (SAURs) and 7 ethylene responsive transcription factors (Table 2). Auxin and ethylene are two of the most important plant hormone pathways and together are involved in nearly every stage of plant development[19].

**Table 1:** Functional clustering of genes disrupted (less than 0.5-fold expression or more than 1.5-fold expression) by BPA treatment. Analysis by DAVID.

Category	Description	Count	Percent disrupted genes	p-value	Fold	Bonferroni
GO:0010033	response to organic substance	28	25.9	6.74E-13	5.0	1.58E-10
GO:0009719	response to endogenous stimulus	24	22.2	3.67E-11	5.1	8.63E-09
GO:0009725	response to hormone stimulus	22	20.4	4.98E-10	5.1	1.17E-07
GO:0009723	response to ethylene stimulus	9	8.3	2.58E-05	7.3	0.006037
IPR001471:	Pathogenesis-related transcriptional factor and ERF	7	6.5	2.65E-05	11.8	0.003522
SPPIR KEYWORD	Ethylene signaling pathway	7	6.5	3.60E-05	11.3	0.003271
UP SEQ FEATURE	DNA-binding region:AP2/ERF	7	6.5	4.45E-05	10.4	0.00541
GO:0003700	transcription factor activity	19	17.6	5.83E-05	2.8	0.006107
GO:0000160	two-component signal transduction system	8	7.4	7.21E-05	7.7	0.016795
SM00380	AP2	7	6.5	7.34E-05	9.4	0.001759
GO:0030528	transcription regulator activity	20	18.5	9.73E-05	2.6	0.010161
GO:0009873	ethylene mediated signaling pathway	7	6.5	1.46E-04	8.6	0.033742
GO:0009755	hormone-mediated signaling	10	9.3	1.82E-04	4.8	0.041818
GO:0032870	cellular response to hormone stimulus	10	9.3	1.82E-04	4.8	0.041818
GO:0045449	regulation of transcription	21	19.4	7.43E-04	2.2	0.160279

**Table 2:** Selected hormone related genes disrupted by BPA. Analysis by DAVID.

Gene ID	Fold	Gene Description
AT5G37260	1.95	Rve2
AT4G38840	1.84	Small Auxin Upregulated RNA 14
AT2G33380	1.78	Caleosin 3
AT4G12550	1.65	Auxin-Induced In Root Cultures 1
AT4G38860	1.64	Small Auxin Upregulated RNA 16
AT4G34760	1.63	Small Auxin Upregulated RNA 50
AT5G62920	1.61	Two-component response regulator ARR6
AT2G18300	1.60	Transcription factor bHLH64
AT3G11410	1.59	Protein phosphatase 2C 37
AT3G20470	1.56	ATGRP-5
AT4G34790	1.56	Small Auxin Upregulated RNA 3
AT5G07580	1.54	Ethylene-responsive transcription factor ERF106
AT1G69530	1.53	Expansin-A1
AT5G43700	1.53	Auxin-responsive protein IAA4
AT5G67190	1.52	Ethylene-responsive transcription factor ERF010
AT2G20880	1.51	Ethylene-responsive transcription factor ERF053
AT5G67190	1.51	Homeobox-leucine zipper protein HAT22
AT4G08040	0.47	ACS11
AT5G52020	0.47	Ethylene-responsive transcription factor ERF025
AT3G23250	0.46	ATMYB15
AT4G17500	0.46	Ethylene-responsive transcription factor 1A
AT1G33760	0.45	Ethylene-responsive transcription factor ERF022
AT1G77640	0.41	Ethylene-responsive transcription factor ERF013

There are several known connections between auxin and ethylene signaling including 1-amino-cyclopropane-1-carboxylate synthase 11 (*ACS11*)[19,20]. ACS11 is an enzyme involved in the biosynthesis of ethylene. It is expressed throughout the plant early in development, but shows tissue specific expression in one month old plants[20]. In response to treatment with auxin, *ACS11* displayed both increased transcriptional expression and an altered pattern of expression. In response to BPA, *ACS11* showed less than 50% expression as compared to untreated plants. This result from transcriptome analysis was confirmed by quantitative PCR (Fig 1). These results indicate that BPA caused changes in gene expression to hormone responsive genes in *A*. *thaliana*.

There was a near concentration-dependent reduction in expression of *ACS11* with about 50% expression at 10–1,000nM BPA and as little as 6% expression at 10,000nM BPA (Fig 2). These results show that the change in gene expression for the hormone responsive gene *ACS11* in response to BPA was concentration-dependent.

### BPA disrupts A. thaliana morphology

**Fig 3:**
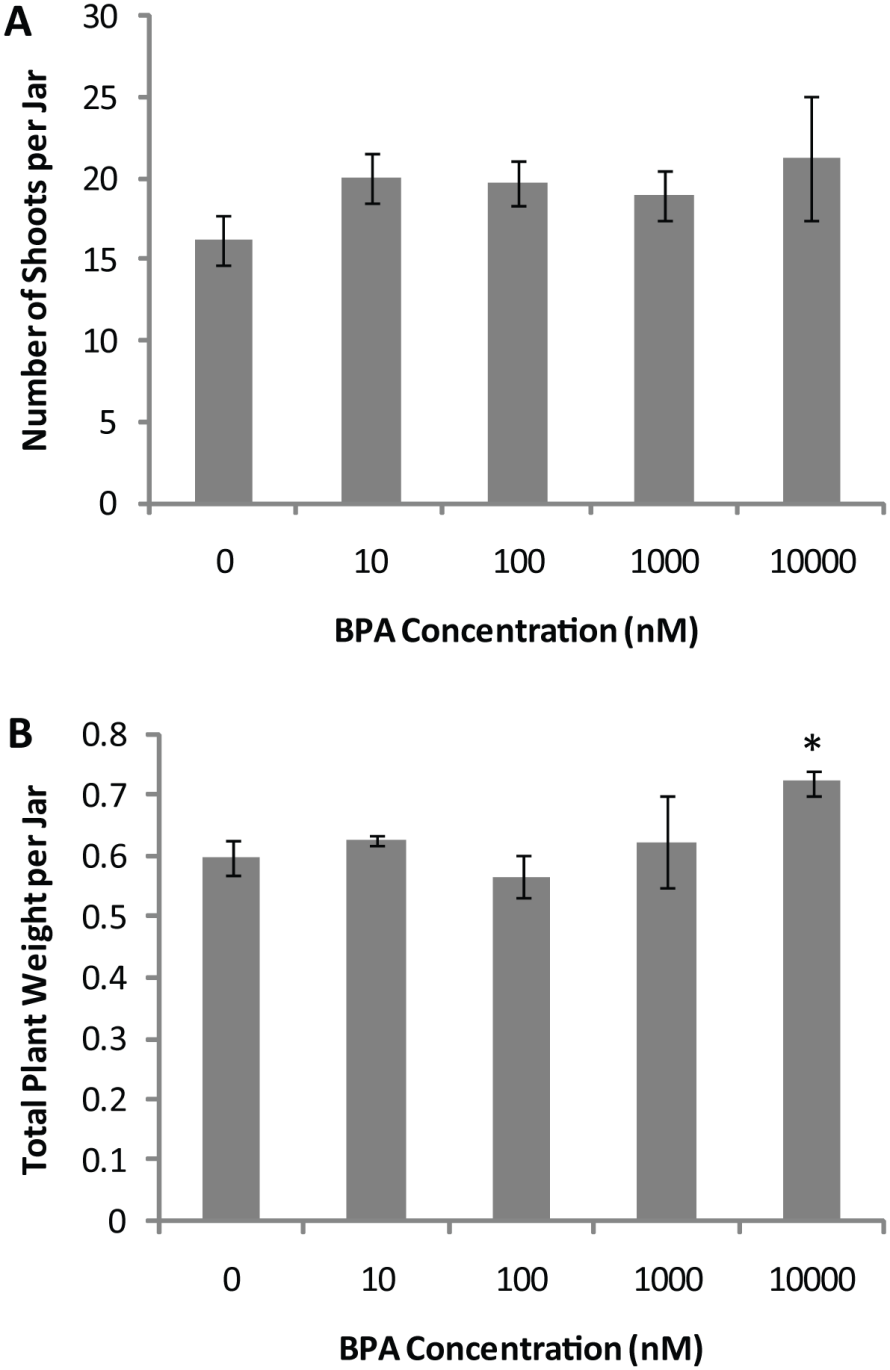
Effect of BPA on gross plant morphology. BPA had no significant effect on the number of shoots produced (A) and had a slight effect on total plant weight per jar (B). Bars represent the average of four jars of plants grown on agar containing BPA at each concentration. Error bars represent standard error. * indicates p-value <0.05 when compared to 0nM BPA treatment.

**Fig 4:**
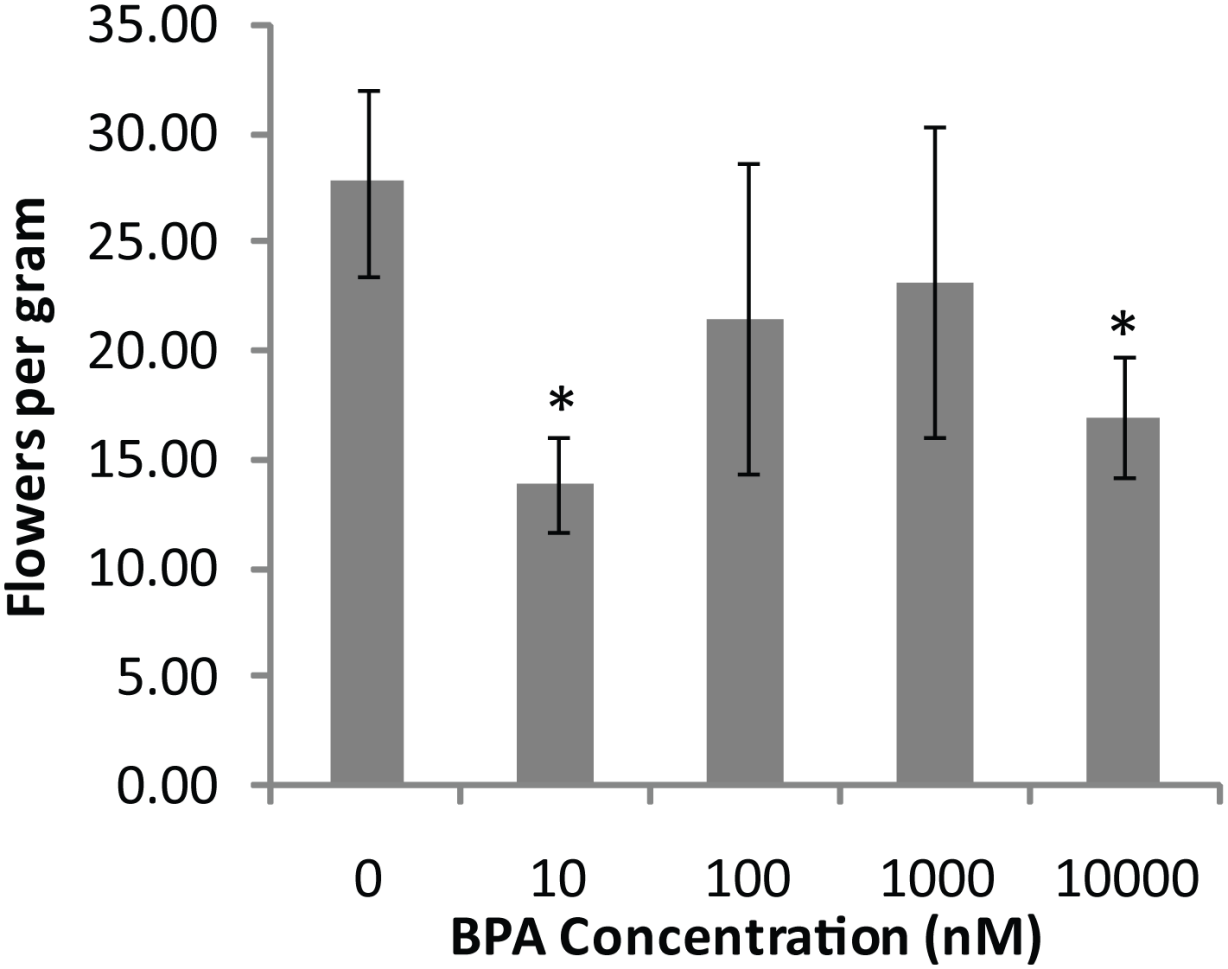
The effect of BPA on plant flowering. BPA may disrupt flowering in *A*. *thaliana*. Bars represent the average of four jars of plants grown on agar containing BPA at each concentration. Error bars represent standard error. * indicates p-value <0.05 when compared to 0nM BPA treatment.

The morphological effects of BPA were determined by growing the plants on agar medium in a range of BPA concentrations (10nM– 10μM). Plants harvested after 26 days of growth showed little significant differences in shoots per jar or total plant weight (Fig 3). This result indicated that BPA concentrations up to 10μM are not lethally toxic to the plants. However, plants treated with BPA had decreased flowering as compared to untreated controls (Fig 4). BPA induced a statistically significant decrease in flowering at both 10nm and 10μM concentration but the decrease was not statistically significant for the intermediary concentrations. These results indicate that the BPA concentrations tested had little effect on gross plant morphology but were able to disrupt plant flowering.

### Disruption of auxin signaling

**Fig 5:**
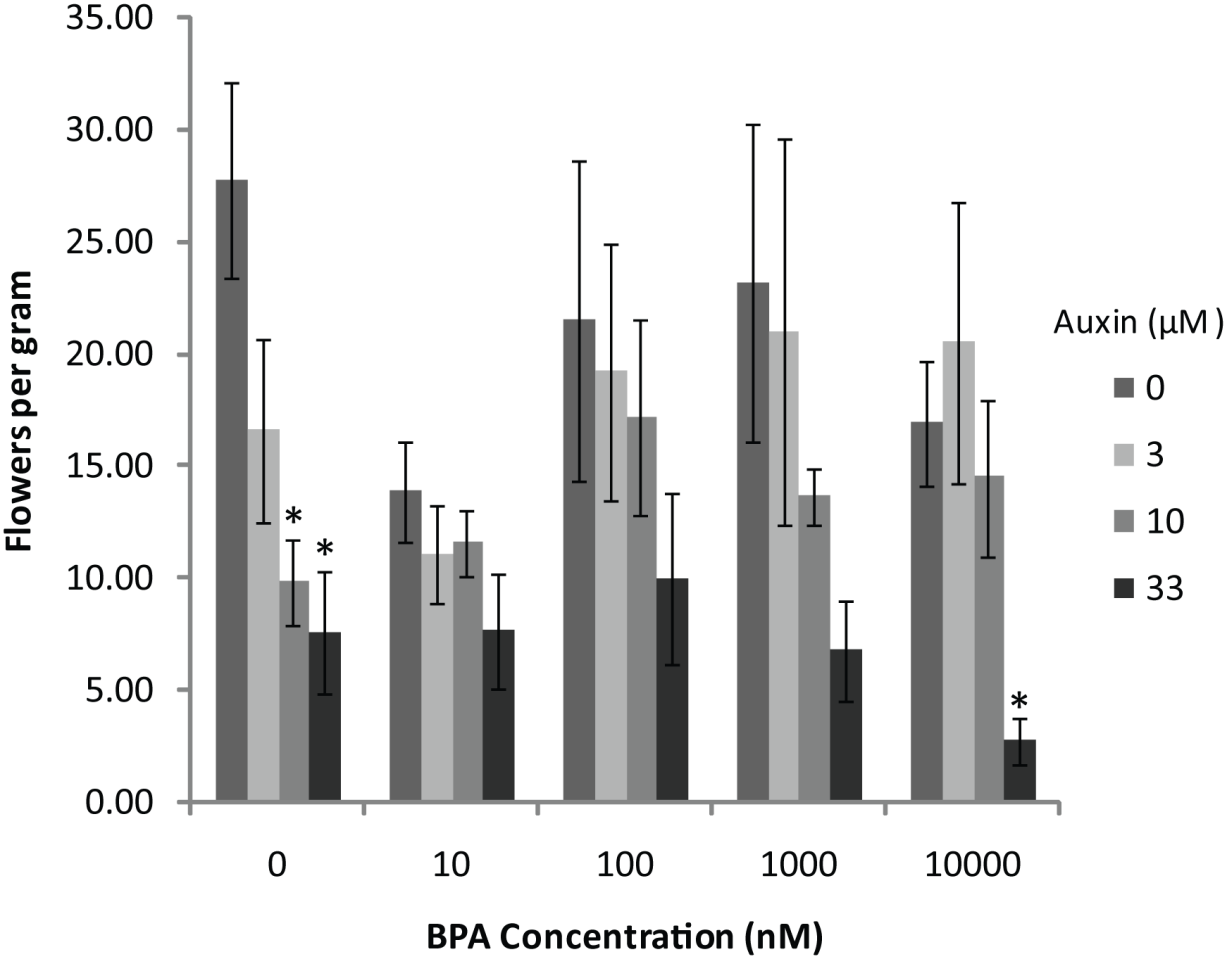
The effect of auxin on plant flowering with and without BPA. Auxin disrupted flowering in *A*. *thaliana* as shown in the 0nM BPA data. The decrease in flowering is not significant when BPA is present except at 10μM BPA and 33μM auxin. Bars represent the average of four jars of plants grown on agar containing BPA and auxin at each concentration. Error bars represent standard error. * indicates p-value <0.05 when compared to 0μM auxin treatment at that BPA concentration.

One possible mechanism by which BPA could disrupt plant flowering is through improper response to the plant hormone auxin. The genomic evidence gathered so far has indicated that auxin signaling could be involved in the BPA response. The effect of BPA on auxin signaling was determined for two reasons. First, many hormone responsive genes showed disrupted expression in the presence of BPA. Second, ACS11, an auxin dependent gene, displayed a concentration-dependent change in expression in response to BPA. Auxin disrupted flowering in a concentration-dependent manner (Fig 5, 0nM BPA bars)[21]. However, plants that are treated with both BPA and auxin showed intermediate levels of flowering (Fig 5). In the presence of BPA, auxin concentrations of 3μM to 10μM did not show the concentration-dependent disruption of flowering that was seen in the absence of BPA. These results indicate both that BPA may disrupt auxin signaling in *A*. *thaliana* and the genomic changes from BPA exposure may have morphological consequences as well.

### General conclusions

The established environmental toxin BPA can be found in concentrations as high as 75μM in untreated landfill leachate^1^. The data presented here show that at much lower concentrations (0.2 μM) BPA can cause changes in gene expression for numerous genes involved in plant hormone signaling. At least one of these genes (*ACS11*) displays a concentration-dependent change in gene expression in response to BPA. The toxin can also disrupt flowering of *A*. *thaliana* and can mitigate reductions in flowering that come from high concentrations of auxin.

BPA is a known mammalian hormone disrupter and this report indicates it is also able to disrupt hormone signaling in plants. More work is necessary to determine whether BPA pollution can disrupt the timing or degree of flowering in the environment. This toxin may interrupt plant-animal interactions including pollinator visits and seed production and these disrupted relationships could in turn affect whole ecosystems. It is imperative that alternatives to BPA that are less environmentally consequential be developed and evaluated for plant and animal toxicity[22].
